# Gut Microbiota and Short-Chain Fatty Acids: Key Factors in Pediatric Obesity and Therapeutic Targets

**DOI:** 10.3390/ijms262311503

**Published:** 2025-11-27

**Authors:** Alin Constantin Pînzariu, Sebastian Marian Leonte, Alexandra Gabriela Trofin, Laura Mihaela Trandafir, Mihaela Moscalu, Lorena Mihaela Manole, Roxana Moscalu, Cristina Iuliana Lazăr, Luminita Georgeta Confederat, Vlad Ionuț Vlăsceanu, Radu Petru Soroceanu, Daniel Vasile Timofte, Dragomir Nicolae Şerban, Ionela Lăcrămioara Şerban

**Affiliations:** 1 Grigore T. Popa University of Medicine and Pharmacy Iasi, 700115 Iasi, Romania; alin.pinzariu@umfiasi.ro (A.C.P.); mg-rom-33565@students.umfiasi.ro (S.M.L.); mg-rom-31141@students.umfiasi.ro (A.G.T.); lorena.manole@umfiasi.ro (L.M.M.); crislazar@ymail.com (C.I.L.); confederat.luminita@email.umfiasi.ro (L.G.C.); vlad-ionut.vlasceanu@umfiasi.ro (V.I.V.); petru.soroceanu@umfiasi.ro (R.P.S.); daniel.timofte@umfiasi.ro (D.V.T.); dragomir.serban@umfiasi.ro (D.N.Ş.); ionela.serban@umfiasi.ro (I.L.Ş.); 2 Blond McIndoe Laboratories, Division of Cell Matrix Biology and Regenerative Medicine, School of Biological Sciences, Faculty of Biology, Medicine and Health, The University of Manchester, Manchester Academic Health Science Centre, Stopford Building, Manchester M13 9PL, UK; roxana.moscalu@postgrad.manchester.ac.uk

**Keywords:** obesity, gut microbiota, gut barrier function, 16S rRNA, SCFAs, pediatric, probiotics

## Abstract

Obesity in the pediatric population has become a public health problem with an increasing prevalence in recent years. The gut microbiota and its metabolites, especially short-chain fatty acids (SCFAs), play vital roles in the development of pediatric obesity. This manuscript aims to highlight the link between the gut microbiota and obesity by examining microbial imbalances, such as altered *Firmicutes*/*Bacteroidetes* ratios and altered SCFA production, especially butyrate, propionate, and acetate. Advances in genomic technologies, such as 16S rRNA sequencing, have enabled personalized treatments and detailed profiling of microbial diversity. This review includes not only preclinical studies but also clinical trials of interventions, such as probiotics, synbiotics, prebiotics, and butyrate supplements. These may modify the microbiota and SCFA levels to reduce obesity and related conditions, such as metabolic syndrome and precocious puberty. Future research should focus on using advanced genomic sequencing techniques to develop new therapeutic strategies, as well as on creating a complex predictive algorithm for assessing metabolic risk in pediatric patients with obesity, an innovation that can be extended to other metabolic conditions.

## 1. Introduction

The incidence of obesity among newborns and children is one of the most important and recent public health problems [1].The World Health Organization has highlighted the fact that in 2022, approximately 160 million children and adolescents aged 5 to 19 years were living with obesity, and in 2024, there were 35 million cases of overweight children under 5 years of age [2]. It is expected that in the coming years, the number of cases of overweight and obese children will increase [1]. In 2050, one in six children and adolescents will be obese [3]. The obesity among children constitute important medical challenges. There are various causes such as dietary, genetic and psychological factors, poor social and economic conditions, sleep hygiene, and reduced physical activity, but also some external environment factors [4,5,6,7,8].

The totality of microorganisms in the digestive tract makes up the intestinal microbiota. It significantly influences the host by being involved in various regulatory processes of the body [9,10,11,12]. The intestinal microbiome is involved in the synthesis of some vitamins (K and several components of vitamin B), the fermentation of fibers and the generation of short-chain fatty acids (SCFAs), the metabolism of lipids and proteins, the decomposition of certain polyphenols, the metabolism of xenobiotics and drugs, and the role of immune protection against various pathogens from food (intestinal barrier) [13,14,15,16,17,18,19,20,21].

Evidence from recent decades has highlighted the close correlation between the microbiome, short-chain fatty acids, and pediatric obesity, characterized by the marked imbalances in bacterial species in obese children. This imbalance between the gut bacteria is named dysbiosis [22].

The purpose of this review is to highlight the close link between obesity in pediatric patients and the gut microbiome, along with the role of SCFAs in obesity and its complications in children, and to present the main methods of prevention and treatment, establishing possible prophylactic measures that may be imposed in the future.

## 2. Materials and Methods

For the purpose of this literature review, a literature search focusing on the articles published in the last 5 years has been performed to look into gut microbiota and short-chain fatty acids. Thus, relevant research studies published between January 2020 and August 2025 were identified through 3 scholarly databases such as PubMed, EBSCO, and EMBASE. In this literature review, 23 relevant studies were studied. Their analysis may lead to the relationship between the microbiome, SCFAs, and pediatric obesity being reinforced. Eligible study designs comprised observational studies (cross-sectional, cohort, and case–control) as well as systematic reviews and meta-analyses, provided that validated diagnostic or screening instruments were applied. These criteria were designed to yield a methodologically homogeneous and clinically relevant evidence base, thereby facilitating a robust synthesis of prevalence estimates, risk factors, and clinical implications of pediatric obesity. To ensure the relevance and methodological precision of the evidence base, exclusion criteria were systematically applied. We excluded articles that did not directly address pediatric obesity—for example, studies focusing exclusively on other medical conditions without an obesity component—as well as editorials, commentaries, letters to the editor, and narrative reviews lacking a systematic methodology. Studies that failed to report data on prevalence, risk factors, or associated clinical outcomes were also excluded. Further exclusions applied to studies published in languages other than English or unavailable in full text, and studies employing inadequate methodology, such as the absence of validated diagnostic criteria or standardized assessment instruments. These criteria ensured the exclusive selection of rigorous scientific studies with comparable data that was relevant to the study objectives.

## 3. Short-Chain Fatty Acid Production

*Bacteroidetes*, *Proteobacteria*, *Actinobacteria*, *Firmicutes*, and *Verrucomicrobia* are considered to be the most important bacterial phyla in the intestinal microbiota, actively participating in the energy metabolism of the host and in the maintenance of homeostasis [23,24].

The genera *Bacteroides*, *Eubacterium*, *Faecalibacterium*, *Alistipes*, *Ruminococcus*, *Clostridium*, *Roseburia*, and *Blautia* play a special role in the production of SCFAs. These are active partners in the digestion process and, through commensalism, ensure the intestinal and systemic morpho-functional integrity through complex and still incompletely elucidated mechanisms. Propionate production is found predominantly in the *Bacteroides* and *Negativicutes genera*. At the same time, Gram-positive bacteria from the *Firmicutes* phylum are involved in the production of butyrate, the most abundant short-chain fatty acid. Other articles mention the major role of *Bifidobacterium* and *Lactobacillus* in the production of SCFAs [25].

As a particular element, in the case of infants and young children, factors may directly modify the production of SCFAs. Breast milk or milk formulas stimulate the multiplication of Bifidobacterium that are involved in the production of SCFAs at very young ages. Due to diversification and progress towards adult life, the number of bacteria will decrease [26]. The microbial population in the intestine of newborns and children is not as diverse as it is in adults. Among the most important representatives mentioned in the literature are *Prevotella corpi, Eubacterium rectale*, and *Roseburia* spp. They ferment dietary fibers with the production of SCFAs that will fulfill numerous roles both locally and systemically. The main biochemical pathways for the production of short-chain fatty acids are similar regardless of whether we are talking about adults, pediatric patients, or overweight animal models [27,28]. Also, luminary pH can influence the production of SCFAs [29]. The diet of children and adolescents significantly impacts the production of short-chain fatty acids. Replacing the consumption of foods rich in saturated fats with foods abundant in unsaturated fats improves the integrity of the intestinal barrier [30]. It ensures a beneficial bacterial balance, reducing inflammation and promoting microbiota activity [31,32].

Fast food and foods rich in lipids influence the biochemical synthesis of short-chain fatty acids. It is well known that children and adolescents, in particular, consume such products, choosing them without taking into account their nutritional information. The regular consumption of fast food among children and adolescents is a predisposing factor for obesity and negatively affects the synthesis of SCFAs [33]. Compulsive eating and lack of dietary discipline in obese pediatric populations maintain a deficit of SCFAs, leading to the emergence of various metabolic disorders that can be perpetuated and chronicized during adult life [34]. A diet rich in fruits and vegetables is an important source of dietary fiber. These fibers are processed by the intestinal microbiome. Carbohydrates are broken down by bacteria into monosaccharides, resulting in hexoses/pentoses and fucose/rhamnose [35]. Regarding the pediatric population, carbohydrates are provided by a diet rich in fiber; this is only applied to children who have started diversification. Carbohydrates are converted into monosaccharides by specific enzymes found in the digestive tract. In newborns and infants who are breastfed, the oligosaccharides in breast milk cannot be broken down by digestive enzymes. Thus, in the colon, certain types of bacteria such as Bifidobacterium break down oligosaccharides into monosaccharides, making it possible to form short-chain fatty acids [36].

Propionate can be synthesized by three main biochemical pathways: the propanediol pathway, the succinate pathway, and the acrylate pathway. In the propanediol pathway, fucose is enzymatically degraded to dihydroxyacetone phosphate (DHAP) and L-lactaldehyde. These intermediates are converted to propane-1,2-diol, which is then converted to propionaldehyde, which is coupled to coenzyme A to generate propionyl-CoA. Propionyl-CoA generates propionic acid through hydrolysis or transfer of CoA. Glycolysis of hexoses/pentoses generates phosphoenolpyruvate (PEP). This is an immediate precursor of pyruvate, but it can be carboxylated by adding a molecule of CO_2_ and converted to oxaloacetate. The acrylate pathway involves the reduction of pyruvate to form lactate. This is converted to lactoyl-CoA, which will be dehydrogenated to form acryloyl-CoA. The latter is reduced to form propionyl-CoA, from which propionate will result. The same pyruvate molecule can be carboxylated to form oxaloacetate, which will ultimately be reduced to succinate. In the succinate pathway, succinic acid is converted to methylmalonyl-CoA, which will generate propionyl-CoA, a precursor for propionate [37].

Pyruvate is a central intermediate in the synthesis of short-chain fatty acids and can generate not only oxaloacetate but also acetyl-CoA. By condensing two molecules of acetyl-CoA, acetoacetyl-CoA is formed, which through successive reduction processes, will generate butyryl-CoA. In the phosphotransbutyrylase pathway, the butyryl-CoA is converted to butyryl-phosphate, which will be converted to butyric acid [38].

**Figure 1:**
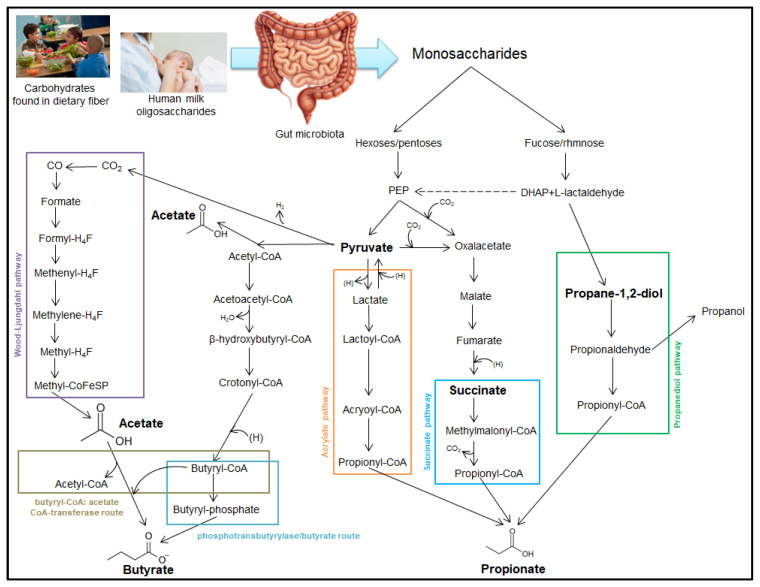
Production of short-chain fatty acids (SCFAs). Phosphoenolpyruvate—PEP; dihydroxyacetone phosphate—DHAP; coenzyme A—CoA; tetrahydrofolate—H_4_F.

The acetate kinase-mediated pathway leads to the transformation of acetyl-CoA into acetate, but it can also be obtained via the Wood–Ljungdahl pathway. This biochemical pathway is used by acetogenic bacteria in the gut microbiome to produce acetate and begins with the reduction of carbon dioxide to formate. This will be converted to formyl-H4F, which will successively pass through reduction to methenyl-H4F, methylene-H4F, and methyl-H4F. The methyl group from the latter compound is transferred to methyl-CoFeSP, which will ultimately generate acetate. It is important to note that butyryl-CoA obtained via the phosphate pathway, under the action of an enzyme, transfers the CoA group to acetate, which will ultimately lead to butyrate. The formation of SCFAs is illustrated in Figure 1 [39].

## 4. SCFAs and Obesity

### 4.1. SCFAs

#### 4.1.1. Physiological Roles of SCFAs in Pediatric Health

Short-chain fatty acids have important roles in maintaining intestinal and whole-body health. The effect are local and systemic, in tissues and organs, through complex pathways [40,41,42,43]. SCFAs ensure the integrity of the intestinal barrier and mediate mucosal immunity at this level, preventing foodborne pathogens from entering the bloodstream and spreading to the systemic level [44]. The regulation of appetite and satiety intervene in glucose homeostasis and may influence insulin sensitivity and immunomodulation [45,46,47,48]. SCFAs help increase insulin sensitivity [49,50]. The increase in butyric acid production and its action on epithelial cells at this level ensures the tightness of the epithelium [51,52].

SCFAs act in various ways and produce various cellular changes that depend on the cell type on which they act. The GPR141 and GPR43 receptors are present at the level of colonocytes and allow for the binding of propionate and acetate. However, enteroendocrine cells also detect this type of metabolites through the same type of receptors. At the cellular level, SCFAs exert inhibitory action on HDAC (histone deacetylase) with functions in regulating the expression of genes associated with cell proliferation and differentiation, immune response, and epithelial integrity. SCFAs can mediate immunosuppression in various immune cells, consequently causing hyporeactivity to commensal bacteria. SCFAs are important metabolites of the gut–liver axis and impact hepatic physiology by triggering the secretion of GLP-1 by enteroendocrine cells, which improves glucose tolerance [53,54].

Short-chain fatty acids can enhance blood–brain barrier function and impact the activity of the nervous system, playing a role in modulating microglia and cytokine activity and facilitating regulation of oxidative stress, metabolic, and neurotransmitter pathways [55,56,57,58,59,60].

#### 4.1.2. SCFAs in the Pathogenesis of Pediatric Obesity

**Figure 2:**
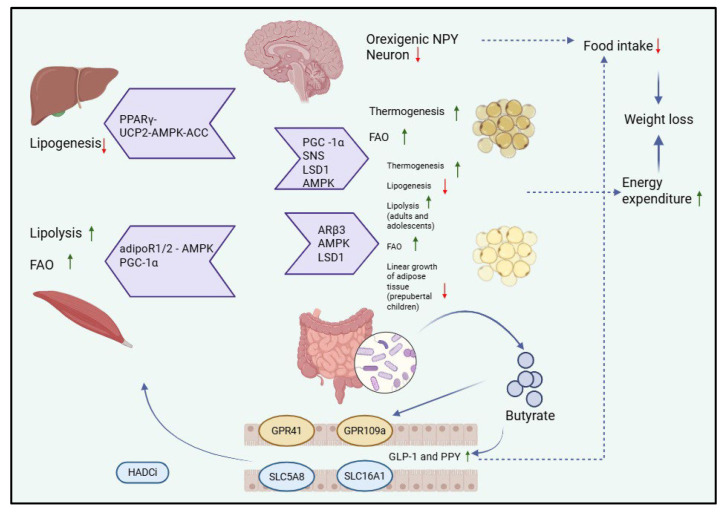
Physiological pathways of butyrate that lead to decreased food intake and increased energy expenditure, through the influence of the brain, liver, striated muscle, and white or brown adipose tissue. Peroxisome proliferator-activated receptor gamma—PPARγ; uncoupling protein 2-AMP-activated protein kinase-acetyl-CoA carboxylase—UCP2-AMPK-ACC; adiponectin receptor 1 and 2—adipoR1/2; peroxisome proliferator-activated receptor gamma coactivator 1-alpha—PGC-1α; fatty acid oxidation—FAO; sympathetic nervous system—SNS; lysine-specific demethylase 1—LSD1; AMP-activated protein kinase—AMPK-ACC; histone deacetylase inhibitors—HADCi; adrenergic receptor Beta 3—ARβ3; G-protein receptor 41—GPR41; G-protein-receptor 109 A- GPR109a; solute carrier family 5-associated 8—SLC5A8; solute carrier family 16-associated 1—SLC16A1.

Short-chain fatty acids are involved in the pathogenesis of obesity through several pathophysiological pathways. Butyrate plays enteroendocrine roles by increasing the production of the hormone GLP-1 and FYY, which leads to a decrease in food consumption with weight loss. Through the action of butyrate on GLP41, GLP99 receptor changes will occur at the level of various organs that will lead to a decrease in food intake and an increase in energy consumption. In muscle tissue, lipolysis intensifies. In the liver, lipogenesis decreases. In white and brown adipose tissue, a decrease in lipogenesis, an increase in lipolysis, and an increase in thermogenesis are observed. In both muscle and adipose tissue, fatty acid oxidation increases. At the brain level, butyrate leads to a decrease in impulses from neurons that regulate appetite and satiety, resulting in the inhibition of neurons that secrete the orexigenic peptide Y, thus reducing the amount of food ingested. SCFAs achieve through these pathways a decrease in food intake and an increase in energy consumption, which will lead to weight loss, and thus, obesity can be treated/prevented [61,62,63,64], as illustrated in Figure 2.

**Figure 3:**
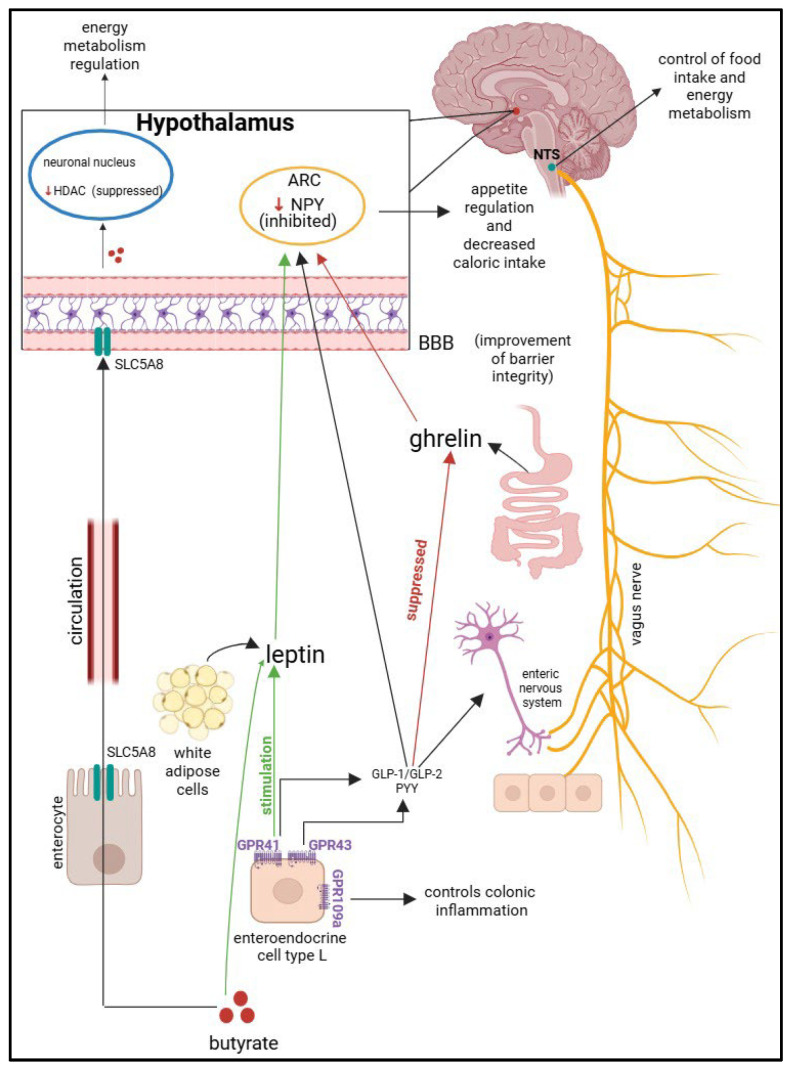
The gut–brain axis pathway. Nucleus tractus solitarius—NTS; arcuate nucleus of the hypothalamus—ARC; blood–brain barrier—BBB; neuropeptide Y—NPY; histone deacetylase—HDAC; solute carrier family 5-associated 8—SLC5A8; G-protein receptor 41—GPR41; G-protein receptor 43—GPR43; G-protein-coupled receptor 109 A (HCAR2)—GPR109a; glucagon-like peptide-1—GLP-1; glucagon-like peptide-2—GLP-2; peptide YY—PYY.

SFCAs act on G-protein coupled receptors (GPCRs), which are represented by GPR41 (FFAR3—free fatty acid receptors 3), GPR43 (FFAR2—free fatty acid receptors 2), and GPR109a (HCAR2—hydroxy-carboxylic acid receptor 2). These receptors are responsible for the actions of butyrate in the body and are found in a wide variety of cells. GPR41 is expressed on adipose cells, peripheral nerves, pancreatic -β cells, enteroendocrine L and K cells, and thymus cells. GPR43 is also found on enterocytes, adipocytes, pancreatic-β cells, enteroendocrine L cells, and some cells of the immune system. Butyrate stimulates GPR41 and GPR43, which will induce the secretion of GLP-1/GLP-2 and PYY; they will interact with the enteric nervous system that will stimulate the vagus nerve and activate the nucleus of tractus solitarius (NTS), which is responsible for regulating eating behavior and energy metabolism. Leptin and ghrelin are two hormones with antagonistic functions involved in the regulation of appetite and satiety. Leptin has the role of reducing the feeling of hunger, and ghrelin to increase it. Leptin is produced by white adipocytes, and ghrelin is secreted by the digestive system. The GPR41 receptor stimulates the leptin pathway, while the ghrelin pathway is suppressed through the action of butyrate-GLP-1. Leptin and GLP-1 will cross the blood–brain barrier and inhibit neurons that secrete neuropeptide Y (NPY) from the arcuate nucleus (ARC) of the hypothalamus, controlling appetite and decreasing food intake. Butyrate is absorbed into the systemic circulation from enterocyte level through the transporter SCL5A8 (solute carrier family 5 member 8). This transporter is the one that transports butyrate to the BBB. Butyrate has an inhibitory action on HDAC (histone deacetylase), which is an enzyme that modulates cellular epigenetic mechanisms, thus regulating the energy metabolism of the organism. SCFAs improve the integrity of the blood–brain barrier. The mechanism described above is illustrated in Figure 3 [61,65,66,67,68,69,70,71,72,73,74]. Recent studies have shown that the gut microbiota can lead to the formation or stimulation of the production of neurotransmitters such as serotonin, dopamine, and γ-aminobutyric acid (GABA). Butyrate leads to increased production of GABA in the hippocampus, which can indirectly modulate appetite and reduce anxiety-related overeating in obese children. Vagus nerve stimulation could also represent a future therapeutic solution for pediatric obesity, by modulating affective state and eating behavior [75,76].

#### 4.1.3. Special Populations: Maternal SCFAs and Neonatal Obesity Risk

The production of SCFAs in pregnant women and their influence on newborns is also a topic of interest. Gestational factors that positively or negatively influence pregnancy and, indirectly, the subsequent production of SCFAs in the mother and child should not be overlooked. In an animal model represented by mice exposed to cigarette smoke, it was found that females presented an altered composition of the intestinal microbiota with a risk of dysbiosis and a lower level of SCFAs [77]. Similarly to adults, a study by Zubcevic and colleagues highlighted that maternal exposure to nicotine from cigarettes during pregnancy can affect the mother’s gut microbiome during pregnancy, with effects on the fetus. There are also articles that address the connection between maternal dysbiosis during pregnancy and the production of SCFAs in the newborn [78,79,80].

### 4.2. Other Metabolites and Their Involvement in Obesity

In addition to SCFAs, a large number of other microbiota metabolites have been observed to be involved in pediatric obesity. The microbiota produces a wide spectrum of metabolites that synergistically or independently modulate the pathogenesis of obesity in pediatric population. These metabolites are represented by bile acids, triglycerides, cholesterol, saturated and unsaturated fatty acids, trimethylamine (TMA), Glycoprotein acetyl, hippuric acid, xanthine and protein degradation compounds. Beyond obesity, these metabolites are involved in a wide variety of diseases [81,82,83].

Branched-chain amino acids (BCAAs) (valine, leucine, and isoleucine) and aromatic amino acids (AAAs) (phenylalanine, tyrosine, and tryptophan) result from the catabolism of ingested proteins. BCAAs, AAAs, and glutamate were found in the study by Liu et al. to be increased in the serum of obese patients, which makes them risk factors for metabolic diseases such as diabetes, insulin resistance, and fatty liver [81]. In the study by Bondia-Pons et al., a higher amount of BCAAs is found in heavier twins, compared to the lighter twins who were enrolled in the trial [84]. In addition to the metabolic risk associated with an increase in amino acids, an associated cardiovascular risk has also been observed, which is produced by the increased inflammatory factors in circulation, hyperglycemia, and hyperlipemia. One of the AAs most associated with obesity is glutamate. It shows a significant increase in obese individuals, compared to lean ones. It is significantly associated with the genera *Blautia, Dorea,* and *Ruminococcus* [85,86].

Several studies have demonstrated a link between certain bacterial genera and the modulation of blood triglyceride and cholesterol levels. *Blautia* has been positively associated with serum saturated fatty acids [87]. *Bifidobacterial* diversity and plasma long-chain TGs have also demonstrated a negative correlation [84]. Two of the most important polyunsaturated fatty acid (PUFA) derivatives are conjugated linolenic acid (CLnA) and conjugated linoleic acid (CLA). These have been negatively correlated with blood lipid profiles, being considered important elements in weight loss. The bacterial species *Bifidobacterium*, *Eubacterium ventriosum*, and *Lactobacillus abundances* are associated with serum concentrations of CLnA and aCLA [88].

Secondary bile acids are formed by the deconjugation of primary bile acids, resulting in over 50 different types of acids. These hydroxylated steroids contribute to the digestion of dietary lipids. They are considered the main factors that establish the connection between the metabolism of the intestinal microbiota and that of the host organism. They can lead to the impairment of energy expenditure, glucose homeostasis, and lipid and carbohydrate metabolism, which can interfere with the weight loss process [81,89]. Trimethylamine (TMA), together with dimethylamine (DMA), has been found to be increased in the urine of obese patients. In obese individuals and pregnant women, an increase in a low-grade inflammation marker, named alpha1-Glycoprotein acetyl (GlycA), has been observed. This compound is associated with insulin resistance and obesity. Hippuric acid has been associated with a decreased risk of developing metabolic syndrome. Xanthines are purine bases in the body, which through degradation by Xanthine oxidase will be converted to uric acid. A decrease in this metabolite has been observed in obese subjects [81,90].

## 5. Dysbiosis in Obesity

In recent years, the link between the microbiota and its involvement in obesity has been increasingly strengthened, with dysbiosis being the implicated factor in the production and perpetuation of energy imbalances within the body [62]. The emergence of an abnormal microbiota as a structure will also produce deviations in the production of its metabolites, especially SCFAs. Thus these fatty acids will be involved in abnormal weight gain [61,62]. the significant relationship between obesity and the changes that occur at the level of the microbiome and the secretion of SCFAs is strongly illustrated by a large number of studies [91,92,93].

We have chosen for this review to present 23 of the most relevant studies that attest to the connection between the three components discussed in this article, obesity, the microbiome, and SCFAs. We have analyzed data on bacterial diversity, the most significant types of bacteria in the microbiome, and the quantitative changes that characterize these bacteria, including the *Firmicutes/Bacteroides* ratio, as well as the quantity and type of SCFAs. We mention that the F/B ratio is a biological parameter that is used to characterize the structure of the microbiome.

Out of the 23 studies analyzed in Table 1, the majority associated two markers with obesity, thereby high F/B ratio and low SCFAs indicate a close correlation between microbiota and obesity. These markers together with molecular biology techniques could be used to characterize the obese pediatric patient and could lead to their inclusion in a personalized therapeutic program depending on the constituents that make up their microbiota.

The variability of the results described by the studies may be due to errors in sample collection or processing; the lack of a standardized model to conduct such a clinical trial; microenvironment differences; and factors related to the patient’s diet immediately prior to biological sample collection, such as the consumption of extremely high-fiber meals, which could lead to a transient increase in SCFAs. The limitations of the studies analyzed in this article are due to the non-standardized procedures for collecting and processing the samples taken, thus amplifying inter-study heterogeneity. In order to further strengthen the connection between alterations that occur at the microbiota level and the obesity, we propose conducting more longitudinal studies, not just cross-sectional studies. The need to develop standardized clinical trial models that can be applied to a large part of the general population can be observed.

### 5.1. Analysis of the Relationship Between Obesity and the Microbiome

Shihan Li et al. [93] wants to demonstrate the association between microbiota and obesity through his case–control study. He studies a group of 99 children aged 7–14 years, divided into two groups, those with normal weight (NG) and those with obesity (OG). The study concludes that Gram-negative bacteria (*Proteobacteria*, *Escherichia coli*, and *Haemophilus*) are increased in the group of obese children (OG), and *Oscillibacter* and *Alistipes* are decreased [93].

The quantitative increase in Gram-negative bacteria in the case of obese children was also observed in the study led by Peter Gyarmati et al. [94], where they studied parameters such as the diversity and composition of the fecal microbial flora, the F/B ratio, and the amount of SCFAs in different weight typologies (normal weight, overweight, and severe obesity). The study involved 83 children aged between 5 and 12 years. The human microbiome is composed of five major phyla, including *Firmicutes*, *Bacteroidetes*, *Proteobacteria*, *Actinobacteria*, and *Verrucomicrobia*, and the study observed that two types changed when the severely obese group was compared with that of normal weight children: an increase in *Proteobacteria* and a decrease in *Verrucomicrobia* were observed in the morbidly obese group. No significant change in the F/B ratio was observed [94].

Although the F/B ratio is variable in the studies described, changes involving both *Firmicutes* and *Bacteriodetes* at the phylum/species level are considered key elements signaling obesity [95,96]. Differentiation between the degrees of obesity led to the emergence of a varied relative abundance of *Actinobacteriota* and *Bacteroidota* microorganisms [97].

Another investigation conducted in Harbin, China, on 99 children aged 5–15 years highlights a decrease in the F/B ratio in the obese group of children, compared to those with normal weight. The decreased amount of *Lactobacillus* and *Bifidobacterium*, together with the relative increase in the amount of *Akkermansia*, were observed in the obese group. Also, the study aims to elucidate which types of bacteria could be used as strains that have a potential to treat obesity. Of the nine strains discovered to have an antiobesity effect and inhibit weight gain in HFD-fed mice, only two strains qualified to be used as markers in antiobesity screening. These are *Lactobacillus* and *Bifidobacterium* [98]. Another therapeutic target, which could be used in the future against pediatric obesity, is *Bacteroides* [99].

According to research conducted by Araujo D.S. et al. [99], the differences between the microbiota of obese and normal-weight children are achieved by beta diversity. In contrast, alpha diversity does not show differences. It was also observed in both groups that the most predominant bacteria were *Firmicutes* and *Bacteroides*, followed by *Actinobacteria* and *Proteobacteria*. One of the conclusions was that the relative abundance of the genus *Bacteroides* was significantly lower in the group of obese Korean children than in the other group, and at the species level, *Bacteroides ovatus* was mentioned as being lower in the obese group. Hence, we can associate the involvement of *Bacteroides* in maintaining a normal weight in children. The F/B ratio was significantly higher in the obese group. No significant differences were demonstrated in the relative abundance of the bacterial species *Firmicutes, Actinobacteria, Proteobacteria*, and *Akkermansia*. The study presented above involved 22 obese children and 24 normal-weight children aged 5–13 years. The fecal samples were processed and analyzed using 16S rRNA gene sequencing using the Illumina MiSeq platform [99].

Xu-Ming Li et al. [100], in their study conducted on 30 normal-weight and 30 obese children (8–12 years old) using the 16S rDNA sequencing method, present findings consistent with those of the previous study, namely that the F/B ratio is high. This is due to the abundant presence of *Firmicutes* bacteria and the decrease in the abundance of *Bacteroides* bacteria in the obese group. *Provotella* was more abundant in the obese group, compared to *Sanguibacteroides*, which was representative of the control group. Other differences in the intestinal microbiota observed in the obese group of children are lower alpha diversity; an increase in *Clostridioides*; and a reduction in bacteria that are considered probiotics, such as *Lactobacillus* and *Bifidobacterium*. Also, a decrease in SCFA-producing bacteria is observed, as well as a decrease in the presence of a bacterium with a beneficial role in the body, such as *Akkermansia muciniphila*. The increase in the *Enterobacteriaceae* family is associated with the presence of metabolic syndrome in children [100]. Similar conclusions on similar groups of children were also made by L F Jiang et al. [101].

The abundant presence of *Bacteroides* in the lean group and the increase in the abundance of *Provotella* in the obese group can also be observed when comparing the microbial flora of obese/norm-weight children with type 1 diabetes. A difference in beta diversity can also be observed between the two groups; however, the F/B ratio is insignificant. Significant increases in fermentation bacteria such as *Prevotella copri*, *Bacteroides stercoris*, *Parabacteroides merdae*, *Holdemenalla biformis*, and *Bifidobacterium angulatum* were found in the obese group. SCFA-producing bacteria (*Anaerotruncus colihominis*, *Clostridium* sp. CAG:167, *Bifidobacterium pseudocatenulatum*, *Eubacterium* sp. CAG:251, *Eubacterium eligens*, *Bacteroides plebius*, *B thetaiotaomicron*, and *B ovatus*) were found with a higher prevalence in the lean group [102].

Even though the increase in *Firmicutes* and the decrease in *Bacteroides* are associated with obesity, the process of losing weight could destabilize this relationship and reverse the percentages, as shown in the study by Ky Young Cho [91]. The study compared two groups with values of normal-weight children. In one group were included participants who lost weight, and there was another group for those who gained weight. In patients who lost weight, bacteria of the genus *Bacteroides* decreased, and those of the genus *Firmicutes* and *Clostridioides* showed an increase. Regarding the group of those who gained weight, *Firmicutes* and *Clostridioides* decreased, and *Romboutsia* was identified as being involved in driving the changes between the pre-intervention and post-intervention stages [91].

As can be identified above, of particular importance in the production of obesity is the increase in the relative abundance of *Firmicutes*, and with a protective role in those with a normal weight, we find *Bifidobacterium* [103]. Insulin could also be a key factor in regulating the microbiota, as important differences were observed between the group of obese children with insulin resistance and the group of obese children sensitive to insulin. Those in the latter group had a much higher F/B ratio compared to those with insulin resistance [104].

Among other elements that are associated with dysbiosis and pediatric obesity, we also find metabolic syndrome, which is an entity that presents variable pathways of production through alterations of intestinal microorganisms [105,106]. This is seen as a risk for pediatric patients, who may develop cardiovascular disease and nonalcoholic fatty liver disease due to it [1]. The factors involved in the production of intestinal dysbiosis have been underestimated and require more studies to be examined [106].

Among the metabolic changes produced by microbiome-induced obesity, precocious puberty is also present in girls [107,108]. In order to influence the hypothalamic–gonadal axis and to produce precocious puberty associated with obesity, the intestinal flora manages to regulate the expression of the Kiss-1/GnRH pathway [108]. Studies have demonstrated a strong correlation between changes in the microbiome and the occurrence of precocious puberty associated with obesity, especially in girls [107,108].

Among the factors recently discovered to be involved in metabolic syndrome and obesity, we can list *Coriobacteraceae*, *Collinsella*, *Collinsella aerofaciens*, *Erysipelotrichaceae*, *Catenibacterium*, and *Catenibacterium* spp., whose relative abundance increased, and *Parabacteroides distasonis*, which showed a decrease [109]. Chonnikant Visuthranukul et al. [92], in their study, carried out a correlation between the relative abundance of some microorganisms and a series of metabolic markers. *Actinobacteria* and *Bifidobacterium* are positively associated with HDL-C, and *Actinobacteria* is negatively correlated with HOMA-IR and fasting insulin. *Lactobacillus* has a positive relationship with adiposity and the occurrence of acanthosis nigricans, frequently associated with insulin resistance [92].

### 5.2. Analysis of the Relationship Between Obesity and SCFAs

#### 5.2.1. Analysis of the Relationship

Regarding the relationship between SCFAs and obesity in the studies presented above, we will report the conclusions of these studies on SCFAs. We note that not all 20 studies presented data on the analysis of SCFAs. Short-chain fatty acids are metabolites produced through the fermentation of dietary fibers by the intestinal microbiome. The main SCFAs are butyrate, propionate, and acetate [61,62].

Shihan Li et al. [93] report that the main SCFAs associated with low levels and obesity are butyrate and isobutyrate. The increased level of caproate was considered a determining factor of pediatric obesity. SCFAs were quantified from peripheral venous blood [93].

In contrast to the previously reported information, the study by Peter Gyarmati et al. [94] showed obesity to be associated with increased fecal levels of butyrate, isobutyrate, and isopentonate. Significant levels of short-chain fatty acids were found in the feces of morbidly obese children, and a progressive increase in the amount of SCFAs was observed from the normal weight group to the obesity groups [94].

Yuanhuan Wei et al. concluded in their study, which enrolled 236 Chinese children, that measurements taken to quantify the amount of fat in a child’s body increased as SCFAs arised. The study highlights that butyric and acetic acids are the most significantly involved in pediatric obesity [110]. Also, the increase in total SCFAs in feces was considered a significant parameter, which can be correlated with obesity in pediatric age [110,111,112].

The previous conclusions are also supported by the study of Heba M. Ismail et al. [102], where it is mentioned that SCFAs were higher in the fece samples of obese subjects, compared to lean ones. In this study, three acids with a key role in obesity are highlighted, which are increased in obese subjects, namely acetic, butyric, and propionic acid [102]. In the study conducted by Jose’ Dio’genes Jaimes et al., the four most important metabolites that were different between the normal weight and obese groups were arabinose, butyrate, galactose, and trimethylamine. The study found that in addition to high levels of butyrate found in the feces of obese children, monosaccharides were also found, and trimethylamine was correlated with poor health [112]. In addition to fatty acids, imbalances in proteins secreted by the microbiome (secretome) are involved in alterations of the flora and the production of dysbiosis, but also in changes in the host immune system, triggering inflammation [109].

As we can conclude from previous studies, the quantitative reduction in butyrate is a cause that predisposes to obesity. The reduction in butyrate secretion has numerous effects that negatively impact the body. Dysbiosis alters bacterial populations, leading to an increase in the *Firmicutes/Bacteroides* ratio, an increase in the number of bacteria in the phylum *Firmicutes*, and a decrease in the number of bacteria in the phylum *Bacteroides*. There is a reduction in the number of lactic acid-producing bacteria of the genus *Lactobacillus*, of those of the genus *Bifidobacterium*, and of those of the phylum *Verrumicrobia*. Dysbiosis is associated with an increase in the number of bacteria in the phylum *Proteobacteria* and of those of the species *Akkermansia*. These quantitative changes lead to a reduction in the production of short-chain fatty acids, as well as a marked decrease in the amount of butyrate.

**Figure 4:**
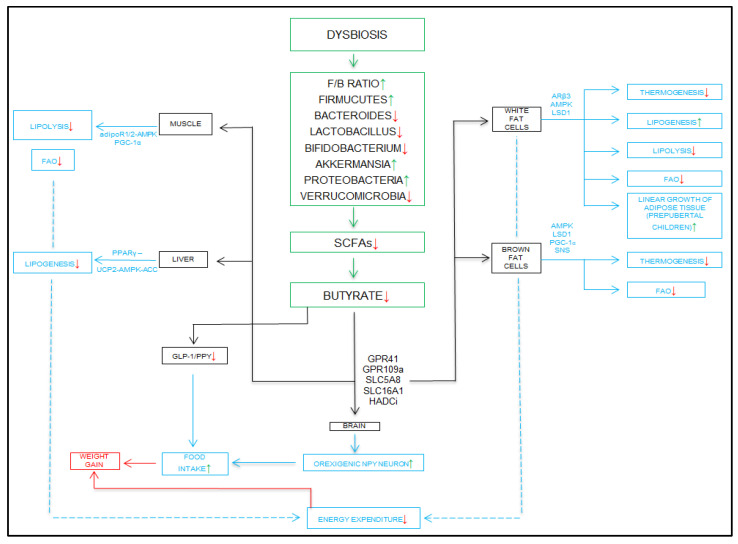
Mechanisms of dysbiosis and decreased butyrate in the production of obesity. Adiponectin receptor 1 and 2—adipoR1/2; peroxisome proliferator-activated receptor gamma coactivator 1-alpha—PGC-1α; peroxisome proliferator-activated receptor gamma—PPARγ; uncoupling protein 2—UCP2; AMP-activated protein kinase-acetyl-CoA carboxylase—UCP2-AMPK-ACC; glucagon-like peptide-1—GLP-1; peptide YY—PYY; Firmicutes/Bacteroides—F/B; short-chain fatty acids—SCFAs; G-protein receptor 41—GPR41; G-protein-receptor 109 A—GPR109a; solute carrier family 5 associated 8—SLC5A8; solute carrier family 16 associated 1—SLC16A1; histone deacetylase inhibitors—HADCi; fatty acid oxidation—FAO; peroxisome proliferator-activated receptor gamma coactivator 1-alpha—PGC-1α; sympathetic nervous system—SNS; adrenergic receptor beta 3—ARβ3; AMP-activated protein kinase—AMPK; lysine-specific demethylase 1—LSD1.

The effect of the decrease in the production of butyric acid is felt at the hormonal and metabolic levels through the numerous types of tissues (muscle, liver, white, and brown adipose) at the level of which metabolic pathways are deregulated. The synthesis of GLP-1 and PYY peptides is reduced successively with the reduction in the GLP-1/PYY ratio. At the hypothalamic level, the feeling of hunger is stimulated, accompanied by increased food intake and weight gain. In muscles, the decrease in butyrate production leads to a decrease in lipolysis and a decrease in fatty acid oxidation. In the liver, a decrease in lipogenesis is observed. In white adipose tissue, the reduction in the amount of butyrate leads to a decrease in thermogenesis, lipolysis, and fatty acid burning. It is accompanied by an intensification of lipogenesis through continuous fat accumulation, while in brown adipose tissue, the biochemical effects are similar. Consequently, the decrease in fatty acid production at the level of these four main tissue types is felt by a reduction in the total amount of energy consumed by the body during a day, which leads to weight gain. Weight gain is the result of both a decrease in the production of satiety hormones and the effect on various tissues in the body responsible for maintaining energy balance and lipid profile homeostasis, as illustrated in Figure 4.

#### 5.2.2. Research Consensus and Controversy

Discrepancies between studies because of the different levels of SCFAs may be due to the type of sample collected (plasma vs. feces), the dietary confounding (e.g., high-fiber diet before sampling), or the degree of obesity (overweight vs. morbid obesity). Plasma levels of short-chain fatty acids better represent systemic availability, while fecal SCFAs better reflect local microbial activity. This distinction needs to be clarified in future studies.

### 5.3. Analysis of the Particular Relationship Between the Oral and Digestive Microbiomes

The child’s oral microbiome is relatively understudied, but several studies attest to its involvement in the production of various pathologies that disrupt the body’s homeostasis. Among the diseases caused by oral microbiome dysbiosis, we can mention obesity, functional constipation, halitosis, and periodontal inflammation [114,115,116,117,118,119]. Halitosis is also a marker that can show systemic damage to the body, especially dysbiosis of the gastrointestinal tract [114].

Thus, we can observe through diseases that damage the oral microbiota and the gastrointestinal microbiota the connection between these two components of the body [120,121,122]. Obesity is considered one of the most important pediatric diseases, which can be caused by both oral and intestinal dysbiosis [99,115,116,118,120,122,123,124,125].

The connection between the bacteria that make up the intestinal microbiome, obesity, and the oral microbiome is established from the first days of life of the newborn and is strongly influenced by his diet [126,127]. Breastfeeding the baby for at least 6 months decreases his risk of developing obesity according to the WHO [128]. Approximately 45% of the bacteria that form the intestinal microbiome are also found in the composition of the oral one, and microorganisms from the oral cavity are translated into the intestinal tract through saliva that is swallowed daily [121].

The way in which the gut microbiome is formed is dependent on several factors, including the type of delivery, whether the baby was breastfed, the composition of the mother’s microbiome, and the baby’s oral microbiome [126,127]. One theory considers that the microorganisms found in breast milk come from the reverse colonization of the mammary gland by bacteria from the newborn’s oral cavity, but more studies are needed to confirm this theory. Breastfeeding, on the other hand, directly influences the oral microbiome, with important changes being found between the oral microbiota of naturally breastfed and formula-fed infants [127].

Several studies have demonstrated the link between the oral microbiome and obesity, thus anticipating the indirect involvement of the gut microbiota [115,116,120,123,124,125]. A higher abundance of *Firmicutes*, *Bacteroidetes*, *Proteobacteria*, *Actinobacteria*, and *Fusobacteria* has been observed in the saliva of children who are obese and overweight, compared to those who have a normal weight [115,122]. A higher value of the F/B ratio is also found in obese youth, both in the digestive and oral microbiomes [115]. Additionally, the connection between the two microbiotas is influenced by ethnicity; *Aggregatibacter* and *Eikenella* were found to have a higher prevalence in the oral microbiome of obese European American children compared to the normoponderal group [124]. *Streptococcus mutans* and *Bifidobacteria* were also characterized as being more abundant in the groups of obese children [99]. Obese girls show a higher abundance of *Lactobacillus* in saliva [116].

Dental caries in very young children have been considered as precursors of dysbiosis and obesity [120,125]. The salivary level of *Firmicutes* can show the state of the gut microbiota in obese children; this aspect is modulated by the presence of caries at an early age [125]. It is necessary to resolve dental caries and periodontal disease in children to prevent oral dysbiosis and obesity [118]. Saliva has been suggested as a future marker for the detection of metabolic syndrome in pediatric patients due to the increased leptin and decreased adiponectin levels that could be demonstrated through it [119].

## 6. Therapeutic Strategies

The epidemic of pediatric obesity represents a public health problem, which is escalating from year to year, and requires rigorous treatment [129]. Existing methods of weight loss, such as changes in lifestyle, diet, and physical exercise, often prove ineffective regarding the pediatric population. It is necessary to develop new therapeutic strategies to facilitate weight loss in children [26,129].

The approach to the pediatric obese patient in the future will require a standardized and personalized methodology depending on the microbiota and the interaction between the microbiota and the host. A whole arsenal of therapies, from supplements containing SCFAs, prebiotics, probiotics, and fecal transplantation to genomic sequencing and the use of multi-“omics’’, can be used for the treatment of pediatric obesity. Such therapies can be combined to achieve a therapeutic regimen personalized for each patient. Also, some of the therapies that will be described below will also have a preventive role, protecting patients at risk of developing metabolic diseases.

### 6.1. Advanced Genomic Sequencing Technologies and Metaproteomics

The integration of advanced genomic sequencing technologies has revolutionized the study of microorganisms, especially the intestinal microbiota. Molecular biology technologies target the bacterial 16S gene [130]. The bacterial genome contains the bacterial 16S gene, which is identified as a specific region of the 16S ribosomal RNA gene. It has a particular function in the translation process, serving as a component of the prokaryotic ribosome. The gene illustrates an important molecular marker in the recognition and classification of bacterial microorganisms [130,131].

Most of the studies cited in this article use 16S rRNA genomic sequencing technology to analyze patient samples [61,62,91,93,94,98,100,110,113,132]. These molecular biology techniques allow for the identification of the microorganism directly from the sample, without the need for its isolation and separate growth [130,133].

Full-length 16S rRNA gene sequencing is an advanced molecular technology that enables better taxonomic classification of bacteria and more precise personalization of therapies for diseases that destabilize the microbiota [134]. In addition to the crucial role of these molecular technologies that have allowed for the characterization and deepening of the human microbiome, we also highlight their involvement in the development of new therapies targeting pathologies that are related to it, such as obesity [135,136]. We can present the efficacy of some adjunctive therapies in obesity, such as the use of certain bacterial strains to create probiotics [137]. We can also create personalized therapies for each patient, for instance the customization of a probiotic according to the composition of the microbiome of each pediatric patient [26,137]. The influence and effectiveness of certain treatments on the microbiome can be observed through these advanced molecular technologies. The effects of antibiotics on bacterial flora can be analyzed in this way, demonstrating their effectiveness [138,139].

Another innovation that requires special attention is the development of a predictive algorithm for obese pediatric children [140]. Obese and overweight children with particular structures of the intestinal flora would be directed more quickly and easily towards personalized therapies that fit their microbiome. The 16S rRNA gene sequencing technique plays a crucial role in implementing this protocol by identifying bacteria at the lowest possible taxonomic level, thereby creating a more precise algorithm [140,141]. This algorithm could also be applied to other metabolic diseases [142]. Such algorithms could also be used to create personalized nutritional diets. These would have a more pronounced impact on obesity than a regular diet, recommended for all patients [143].

Metaproteomics is a term that summarizes a large-scale study of all proteins produced by a bacterial ecosystem. Because of these techniques, we can analyze bacterial proteins both structurally and functionally. We can characterize the interaction between them, their localization, and the changes that occur in the dynamics of some proteins and also quantify their abundance in the bacterial community [144]. Proteins are the main elements of structure, signaling, and interaction of microorganisms. They very clearly trace the bacterial phenotypes that make up the intestinal microbiome [145]. It has been recently demonstrated that the functional profile of the microbiota provides more coherent information than taxonomic classification [146].

Genomic sequencing is used to identify and classify the constituents of the gut flora, but it does not provide information about the dynamic interaction of bacteria in the community nor can it provide information about the relationship between the microbiota and the host genome. Metaproteomics can be used to characterize the physiological and metabolic effects of the gut microbiota on the host [146]. It can be demonstrated by tracking interactions of the gut microbiota in certain host diseases, such as metabolic diseases or inflammatory bowel diseases [147,148]. To have a clearer picture of the microbiome and to prevent gaps that are interposed between the taxonomic structure and the genomic potential, it is necessary to interconnect multiple ‘omics’ such as metagenomics, metatranscriptomics, and metabolomics. Thus, further research should be standardized, and also long-term studies and elements of multi-omics for sample analysis should be included [146].

### 6.2. Diete and Exercise

Diet is one of the first methods applied in the classic treatment of obesity [149]. This is an extremely important factor because a balanced diet can lead to obesity prevention. An element that must characterize a healthy diet is the abundance of fiber in the diet, since this will produce an increase in the amount of SCFAs in the colon, favoring the integrity of the intestinal mucosa [35]. Together with fiber, food polyphenols are extremely important in stimulating the microbiota and producing butyrate through the fermentation process, maintaining the human body in a state of homeostasis [150,151].

Creating personalized diets for the intestinal microbiota presents more benefits than a classic diet. Such personalized diets can be created [149,151]. The VLCKD (very low-calorie ketogenic diet) has proven effective in adults for treating obesity. For the pediatric population, it has been observed that if the mother is engaged in such a diet during pregnancy and breastfeeding, the newborn will develop a diverse microbiota, being mainly composed of bacteria that secrete SCFAs. The physiological mechanisms by which these changes are achieved have not yet been elucidated [151]. A-CDR (adjusted-energy-restricted dietary pattern) is an adjunctive diet in the treatment of pediatric obesity, which has proven its effectiveness. After 12 weeks, children significantly lost weight and their microbiota changed, resulting in increased production of SCFAs. The changes that characterized the microbiome were an increase in the abundance of *Bifidobacterium* and a decrease in *Bacteroides* [149].

The Mediterranean diet is an effective approach to combating obesity, as it reduces indicators of obesity, particularly body mass index [152]. A possible involvement of this type of diet in the modulation of the genetic predisposition to obesity has also been observed [153]. The diet with β-glucan helps stimulate butyrate production [154]. β-glucan is a fiber found in whole grains, bakery products, fruits, and vegetables. β-glucans from oats that have been treated with enzymes are also useful in infant milk formulas, contributing to the diversity of their microbiota [155,156]. Recent evidence suggests that omega-3 fatty acids improve intestinal dysbiosis by increasing the presence of beneficial bacteria such as *Bifidobacterium* and *Lactobacillus*, which apparently promote the production of short-chain fatty acids [31,32]. Also, fermented foods such as yogurt, kefir, vegetables, and other fermented foods rich in probiotics contribute beneficially to the production of SCFAs due to their anti-obesity properties [157,158,159].

Physical activity is defined as another essential component in the treatment of obesity along with nutritional diet [160]. Aerobic exercises can be considered a factor in improving inflammatory markers, reducing body fat mass, and lowering fasting insulin levels [161]. Adding resistance exercises to maintain muscle mass leads to improved cardiometabolic health in obese children [162]. Physical activity can be a valuable non-pharmacological therapy for the prevention and/or treatment of obesity in children and adolescents, with notable and recognized benefits for both the development of the intestinal microbiota and the locomotor system [91,92,163]. Athletes and people who perform regular physical activity have a higher number of bacteria producing short-chain fatty acids in the intestinal microbiome. Children who consistently practice physical exercise increase the diversity of their intestinal microbiota, which contributes to the synthesis of short-chain fatty acids and the prevention of weight gain [164,165].

### 6.3. Supplements Containing SCFAs

Butyrate is the main SCFA involved in obesity; therefore, numerous studies have been conducted for its therapeutic effects [35,166,167,168]. Due to the roles of butyrate, especially those that modulate the immune system, both systemic and intestinal, through anti-inflammatory, antioxidant, antiproliferative mechanisms, it could be used in the future in the treatment of digestive and extradigestive diseases [150,166,167,169].

Butyrate supplements are being studied in terms of both obesity and gastrointestinal diseases, such as IBD (inflammatory bowel disease) and CRC (chronic rectocolitis) [35,166]. They have proven effective in the treatment of early diagnosed IBD in children due to the immunomodulatory roles of butyrate. Following the administration of 150 mg of butyrate for 12 weeks, a decrease in systemic inflammation was observed. Fecal calprotectin also decreased drastically after the administration of the supplements [166]. Butyrate also has effects in modulating glucose metabolism; therefore, it can also be administered to correct the consequences of obesity, such as metabolic syndrome [167]. Regarding obesity, butyrate supplements have produced a decrease in BMI and in the inflammation associated with obesity [167,168]. The administration of butyrate also strengthens the BBB (blood–brain barrier) axis [150].

The mode of administration of butyrate is either orally, through supplements of calcium/sodium butyrate, or in the form of an enema, where its local action reduced levels of pain and inflammation. Propionate and acetate are other SCFAs, less well documented than butyrate, but which are considered to be involved in the therapy of obesity in the future. SCFAs are also considered to be extremely safe to use, which makes them extremely useful future therapies [35].

Certain dietary supplements and medications can influence the amount of SCFAs, achieving therapeutic effects on obesity. A recent study demonstrated that metformin can alter the gut microbiome but can also increase the amount of SCFAs in the circulation, which may be beneficial for patients with obesity [170]. It is recommended that the obese pediatric population with both parents suffering from type 2 diabetes take metformin to prevent the developement of type 2 diabetes [171]. It is recommended to conduct extensive research to elaborate the link between SCFAs and metformin. It might be used preventively by the pediatric population at risk of developing obesity. Melatonin is another old substance that is being discovered to have new properties. It can lead to an increase in SCFAs, improving sarcopenic obesity in the elderly [172]. Future research and studies need to be carried out to demonstrate its usefulness in the pediatric population. A study conducted on HFD (high fat-diet) mice presents the effects of phospholipids from crab eggs, Portunus trituberculatus (Pt-PL), as being antiobesogenic. The study demonstrates that there is a significant decrease in weight, but also in the F/B ratio, and an increase in SCFA in feces. Pt-PL may be used in the future as an effective dietary supplement in the prevention or treatment of obesity [173].

In addition to the importance of SCFA supplements for the treatment of obesity, these compounds have also been shown to be effective in other diseases, such as cardiovascular diseases. A diet supplemented with butyrate slowed the development of atherosclerotic plaques in mice that were deficient in apolipoprotein E (apo-E). Butyrate has been described in numerous studies as a blood pressure regulator and may be used in the future as a dietary supplement that prevents the development of hypertension [174]. They also demonstrate their contribution in infantile neuroprotection [175]. Inflammatory bowel diseases can use butyrate as an adjunvant therapy to reduce inflammation and restore the intestinal barrier but also to maintain the disease in remission [176]. In patients with ulcerative colitis, butyrate supplements produced a decrease in inflammatory markers and a regulation of the circadian rhythm, improving the quality of sleep of the treated patients [177]. Improvement effects of diabetic retinopathy were observed in experimental mice, after they were administered sodium butyrate [178]. SCFA supplements have also demonstrated efficacy in suppressing the progression of endometriosis in experimental animals [179]. SCFAs are also involved in the occurrence of neurodegenerative diseases, such as Alzheimer’s disease. Dietary supplementation with high levels of butyrate and acetate could prevent the occurrence of the disease [180]. A combination of niacin and butyrate could be used to prevent Parkinson’s disease [181]. Kawasaki disease is a vasculitis that affects children, and a recent study showed a possible link between SCFA-producing microbiota and the development of the disease, suggesting that butyrate supplements could be used to prevent it [182].

### 6.4. Fecal Transplantation

The use of this treatment is controversial, as it did not demonstrate effects on weight loss alone. However, a decrease in abdominal adiposity was observed in patients who participated in the study. The post hoc study revealed a transient improvement in metabolic functions [183]. It is suggested that fecal transplantation can lead to increased production of SCFAs in the colon, thereby reducing pain and improving symptoms of the gastrointestinal disease [35]. Non-invasive intestinal microbiome transfer can be performed using encapsulated material for the treatment of obesity [184]. Fecal transplantation could represent a useful therapy in terms of obesity, but more studies are needed to demonstrate the efficacy of this treatment [183].

Although fecal microbiota transplantation (FMT) is still limited in the context of obesity, recent studies have demonstrated that fecal transplantation is a promising therapy that can be used to treat over 85 diseases [185]. It has shown remarkable efficacy in treating recurrent *Clostridium difficile* infections [185,186,187]. Among the diseases in which it can be used are diseases caused by intestinal dysbiosis, diseases associated with antibiotic-induced dysbiosis, inflammatory bowel diseases, and metabolic diseases [185].

The success of this therapy is also limited by several factors related to the donor and recipient, such as the composition and diversity of the intestinal microbiota, the immune system, and factors related to the host’s genetics. Limitations are also brought about by the study protocols, which do not precisely mention the criteria for inclusion and exclusion of donors. The operation of processing fecal matter is ambiguous, and the amount of fecal matter and the diluent used are not specified. Thus, it is difficult to reproduce a study that focuses on fecal transplantation, and standardization of clinical trial protocols is necessary to obtain studies that can be replicated more easily [185,186,187].

The safety and acceptability of this treatment are discussed; fecal transplantation is considered favorable in terms of safety. The probability of transmitting a bacteria or virus that harms the host through fecal transplantation is very low. Possible adverse effects are mostly minor effects such as diarrhea, nausea, vomiting, cramps, and abdominal pain; more severe reactions are rare but can lead to death [185,187]. Two alternatives to FMT are also being explored. They are represented by bacterial community transplantation or virome transplantation. The virome is obtained from fecal filtration and could represent an innovative therapy for certain diseases [187].

### 6.5. Prebiotics, Probiotics, and Synbiotics

The use of supplements, such as prebiotics and probiotics, to address pathologies affecting the intestinal microbiome is being studied with interest, as shown in Table 2. These supplements are considered potential, inexpensive, and effective therapies for the future [35,188]. Prebiotics are non-digestible carbohydrate products that modulate the intestinal microbial flora to achieve a symbiosis between bacteria and the host organism, providing benefits primarily to the host [189]. Probiotics are live microorganisms that, when administered in the appropriate amount, improve the health of the host [95]. Symbiotics have the same properties as prebiotics and probiotics, leading to longer survival of bacteria in the digestive tract, but are considered to be more effective than the other two supplements [188].

Prebiotics are considered an adjuvant therapy in the treatment of obesity that improves metabolic markers in pediatric patients [196]. One of the theoretical mechanisms of action of prebiotics is represented by the modulation of enteroendocrine function. An increase in circulating levels of glucagon like peptide-1 (GLP-1) and anorexigenic peptide YY (PYY) was produced by SCFAs formed after the administration of prebiotics [197]. The increase in endogenous production of glucagon like peptide-2 (GLP-2) plays a role in the restoration of tight junctions of the intestinal barrier, which are responsible for its integrity [195]. Prebiotics achieve an increase in SCFAs in patients suffering from obesity. In terms of reducing BMI and weight loss, prebiotics have had modest results [168]. Inulin is a prebiotic supplement that promotes microbiota diversity and healing of intestinal dysbiosis. It improves the mechanisms of action of the intestinal microbiome, promoting an increase in the abundance of *Bifidobacterium* and several butyrate-producing bacteria [191]. Prebiotics are future therapies against obesity, but they must be individualized, since not all prebiotics are suitable for all patients [26,191].

Probiotics are supplements that, because of live microorganisms, can be used to change the composition and metabolites of the intestinal microbiome but also to modulate antioxidant levels and neuroinflammation [193]. They have the property of preventing obesity. It has been shown that, when administering probiotics between 0 and 3 years, at preschool age, children have a much lower risk of being overweight and obesity [129]. A series of probiotics have been observed to produce positive effects on the complications of obesity, fatty liver, and insulin resistance [95]. Thus, the administration of supplements containing *Bifidobacterium breve* BR03/B632 for 8 weeks led to improved insulin sensitivity in the obese pediatric population [137]. *Bifidobacterium*, *Bacteroides*, and *Lactobacillus* are SCFA-producing bacteria; therefore, they are considered innovative treatments for pediatric obesity [26,190]. *Bifidobacterium breve* also has antimicrobial properties, which prevent the multiplication of human pathogenic microorganisms, becoming an even more important therapy for obesity [190].

Studies have shown that symbiotics lead to alterations in the microbiome structure and to a reduction in BMI. The alterations in symbiotic treatment are the increase in *Prevotella copri*, *Coprococcus eutactus*, and *Ruminococcus* spp. A decrease in the F/B ratio was also observed in the group of patients who were administered symbiotics for 12 weeks, compared to the placebo group [194]. These supplements are also administered for the treatment of SIBO (small intestinal bacterial overgrowth). SIBO is frequently associated with obese pediatric patients, in which it induces the appearance of metabolic syndrome [192]. Some probiotics and symbiotics have been associated with a decrease in BIM and blood lipids, but future studies are needed to strengthen these claims [188]. Prebiotics, probiotics, and symbiotics are currently considered controversial therapies. However, in the future, they are expected to be highly effective and low-cost treatments against obesity; however, more studies need to be performed [95,167,188,189,193].

### 6.6. Implementation Challenges

The challenges of implementing probiotic regimens in the pediatric population are variable. Among the barriers to adherence to probiotics, we mention age; older children tend to have lower adherence to treatment compared to younger children. Probiotic formulations that have a pleasant taste and an attractive shape/color should be used, such as probiotic formulations in chewable tablets or fruit-flavored sachets that can improve adherence to treatment. Formulas that have an unpleasant taste should be avoided. The long duration of treatment can lead to a decrease in adherence over time to treatment, as well as in the case of complex regimens, where probiotics must be administered several times a day. The child’s condition, if they vomit or feel sick, can lead to interruption of the therapeutic regimen. It has been observed in some diseases, such as gastroenteritis, that adherence is higher to treatment in a patient with a more severe form of the disease. Also, an inappropriate attitude of the parents and high costs can lead to the cessation of probiotic regimens. We must inform parents appropriately about the usefulness, the method of administration, and the attitude they should have in relation to these supplements and offer them some sustainable options in terms of costs [198,199,200].

## 7. Clinical Trial Progress and Future Challenges

Studies conducted on adult patients have suggested some connection between the occurrence of digestive diseases and the production of SCFAs. The health of the microbiome, the number of butyrate-producing bacteria and the occurrence of Crohn’s disease or ulcerative colitis are correlated [201,202,203,204,205]. In some situations, a reduction in the production of SCFAs has also been found in adult patients diagnosed with irritable bowel syndrome [206,207]. The decrease in the level of butyric acid may be a risk factor for the occurrence of colorectal cancer. Studies conducted on animal models and humans have highlighted the fact that butyrate promotes colorectal carcinogenesis. This aspect has raised a butyrate paradox in front of the medical community. It has been appreciated that a low dose of butyrate stimulates tumorigenesis, while a higher dose has an inhibitory effect [208]. Some identified studies also suggest that butyrate serves a dual function in the pathogenesis of colorectal cancer, as it maintains the physiology and integrity of colonocytes [209,210]. Through the process of butyrate oxidation, colonocytes remain functional, which contributes to the morphological and functional integrity of the intestinal mucosa. Butyric acid can induce apoptosis in cancer cells and limit tumor spread in the body [211,212,213,214].

It should also be noted that SCFAs can regulate the production of neurotransmitters, contributing to the improvement of symptoms related to ASD, because of the extensive interaction within the gut–brain axis in which microbiota metabolites actively participate [215,216,217]. In a study that included 302 Bulgarian children diagnosed with ASD, it was found that the alteration of the intestinal microbiota by modifying the *Firmicutes*/*Bacteroidetes* (F/B) and *Actinobacteria*/*Proteobacteria* (A/P) ratios may contribute to neurodevelopmental disorders. These ratios could serve as potential biomarkers for the diagnosis of ASD [218]. A suggestive study conducted on a sample of 248 children managed to draw a certain connection between the internalizing and externalizing behaviors of preschoolers aged between 3 and 5 years with the levels of SCFAs taken from the stool [219]. Some studies try to identify a relationship between SCFAs and schizophrenia. SCFA deficiency may be involved in the occurrence of depression and anxiety, impaired cognitive function with lower intellectual performance, mood disorders, and age-related neurodegenerative disorders such as Alzheimer’s or Parkinson’s disease [220,221,222,223,224,225]. The link between the production of SCFAs and the occurrence of neurodegenerative diseases is complex and extensive, not yet fully elucidated [226,227]. An implication of SCFAs in the pathophysiological mechanism of Huntington’s disease has also been discovered [228]: the levels of short-chain fatty acids in the plasma of patients with ADHD are lower than those in undiagnosed patients, an aspect that also depends on the antibiotic medication followed, age, or the use of other stimulants [229]. The low level of SCFAs quantified in the feces of children with ADHD could constitute an early detector for ADHD. Acetic acid together with propionic acid could play the role of biomarkers for the severity of the disease [230].

The progress achieved through clinical trials is remarkable; numerous pathological entities in both adults and pediatric populations have been associated with SCFAs [229]. This will allow the study of new pathophysiological pathways and new therapies targeting these diseases in the future. Among the future challenges, we point out the development of standardized, longitudinal studies that spread over an extended period of time and that can be easily reproduced. In the future, SCFAs could play the role of biomarkers for numerous diseases, such as cardiovascular, neurodegenerative, inflammatory bowel, metabolic, and chronic kidney diseases, as well as obesity. It might offer general information about a homeostatic imbalance, rather than serving as a specific biomarker for a particular pathology.

Given the important involvement of SCFAs and the gut microbiota in pediatric obesity, as well as in other pathologies, future studies should address the following objectives. ‘High-priority’ goals should be to develop age-specific SCFA reference ranges for children and to validate 16S rRNA sequencing as a diagnostic tool for obesity risk. ‘Long-term’ objectives should explore the gut–oral–microbiota axis in pediatric obesity and test the efficacy of fecal microbiota transplantation (FMT) in adolescents with severe obesity. Future studies should also emphasize interdisciplinary collaboration. They should integrate microbiomics with pediatric endocrinology to study the interaction between gut microbiota and puberty. Collaboration with nutrition science should be carried out to create personalized high-fiber diets, as well as collaboration with gastroenterologists, neurologists, and psychiatrists to target diseases with digestive and neurodegenerative determinants.

## 8. Conclusions

Following this review, we aimed to strengthen the link between obesity, microbiome and SCFAs. The major impact of this study the investigation into the roles of short-chain fatty acids in obesity. SCFAs have demonstrated involvement in the pathophysiology of obesity, having a key role in signaling weight gain in pediatric patients. Their role as a marker is associated with their increase in feces, especially butyrate. Butyrate is one of the most important SCFAs and a future effective therapeutic target.

We can highlight the dysbiosis of the intestinal microbiome as a primary factor in the imbalance caused by the secretion of SCFAs, thereby leading to the onset of pediatric obesity. Intestinal dysbiosis can be recognized by a series of elements present in the intestinal microbiome, such as quantitative changes brought about by the *Firmicute* and *Bacteroides* species. Although studies vary, the majority theory states that an increase in *Firmicutes* and a decrease in Bacteroides, as well as an increase in the F/B ratio, would characterize overweight and obesity. *Bifidobacterium* and *Lactobacillus* are bacterial species whose anti-obesity activity has been demonstrated, and they are expected to be included in future adjuvant therapies in the treatment of obesity. This article also presented the link between the oral and intestinal microbiome and obesity, but more elaborated studies need to be conducted.

Therapies that target the microbiome and SCFAs to stop pediatric obesity were described in this study. Among the innovative therapeutic strategies that will improve the treatment of obesity are advanced molecular biology techniques targeting the s16 rRNA gene and the metaproteomics. These techniques enable a more accurate taxonomic classification of bacterial species and also an analysis of their interactions in dynamics. They lead the development of personalized therapies tailored to individual patients.

Thus, future directions should focus on integrating advanced genomic sequencing technologies and metaproteomics for microbiota characterization, making a significant contribution to the fields of molecular biology and precision medicine. Additionally, the development of a predictive algorithm for metabolic risk assessment in obese pediatric patients represents an innovation that can be applied to other metabolic diseases.

Pediatric obesity is a topic of interest that needs to be further studied. Therefore, future studies are needed to understand both the mechanisms of its production, involving the microbiome and the production of short-chain fatty acids, as well as the development of innovative therapies against this pathology.

## Figures and Tables

**Table 1:** Gut microbiota and dysbiosis in childhood obesity.

Authors	Study Type	Main Objective	Outcome	Integrative Interpretation	Key Limitation
Ky Young Cho et al. [91], 2021	Observational and intervention (obese children, 7–18 years, 2-month weight-change program)	To investigate changes in gut microbiota composition, richness, and predicted functional profiles in obese children undergoing lifestyle modification (diet + physical activity).	Obese children showed dysbiotic baseline vs. controls; in the “fat loss” group after intervention: altered composition and predicted functional pathways (e.g., nitrate reduction, aspartate superpathway) though diversity changes were limited.	Lifestyle modification in childhood obesity can modify microbial composition and functional potential, suggesting microbiota plasticity and a pathway by which behavioral change may mediate metabolic risk.	Short intervention (2 months) with modest sample and no randomized control; predicted functions (16S) rather than shotgun/functional assays limit causal inference.
Chonnikant Visuthranukul et al. [92], 2022	Cross-sectional study (obese Thai children 7–15 years)	To compare the gut microbiome of obese children vs. healthy controls and examine associations with lifestyle activity, adiposity, and metabolic profiles.	Obese children had lower abundance of *Bacteroidetes* and *Actinobacteria*, altered beta-diversity; lifestyle and adiposity metrics correlated with microbiota composition.	The link between lifestyle behavior, adiposity, and gut microbiota in obese youth highlights a behavior–microbiome–metabolism axis, emphasizing that modifiable habits shape microbial profiles and hence metabolic risk.	Cross-sectional design cannot establish causality between lifestyle, microbiota, and obesity.
Li S. et al. [93], 2025	Observational study (children with obesity vs. normal weight)	To evaluate associations between gut microbiota composition and fecal SCFA concentrations in obese children.	Obese children showed depleted butyrate-producing bacteria (e.g., *Oscillibacter*, *Alistipes*) and elevated Gram-negative bacteria (*Proteobacteria*, *Escherichia coli*), with lower plasma butyrate/isobutyrate and higher caproate levels linked to obesity.	The depletion of butyrate- and propionate-producing bacteria indicates reduced fermentative capacity and energy imbalance. The strong correlation between SCFA alterations and metabolic markers supports the microbiota–SCFA–obesity axis as a key pathogenic mechanism in childhood obesity.	Single time-point observational design; diet/medication not tightly controlled; fecal SCFAs are correlative and may not reflect systemic exposure.
Gyarmati P. et al. [94], 2021	Cross-sectional study (83 children, 5–12 years, normal weight vs. overweight/severe obesity)	To evaluate microbial diversity, F/B ratio, and SCFA levels by weight classification.	Increased *Proteobacteria*, decreased *Verrucomicrobia*, no significant F/B ratio change; dysbiosis associated with severe obesity.	Higher *Proteobacteria* abundance with obesity severity implies inflammation-driven dysbiosis, highlighting that BMI alone does not predict microbial shifts.	Modest sample (n = 83) and cross-sectional design limit generalizability and causal inference; potential dietary and socioeconomic confounding.
Petraroli M. et al. [95], 2021	Systematic review (children and adults)	To review microbiota, F/B ratio, and SCFA roles in obesity.	Increased F/B ratio, reduced *Bacteroides*; SCFAs regulate energy metabolism in obesity.	Evidence across age groups confirms SCFA-mediated energy regulation; inconsistent F/B ratios limit its utility as a biomarker.	High heterogeneity (populations, sequencing methods, obesity definitions) and publication bias; reliance on F/B ratio oversimplifies community structure.
Indiani C.M.D.S.P. et al. [96], 2018	Systematic review (children)	To analyze F/B ratio and bacterial changes in childhood obesity.	Increased F/B ratio, reduced *Bacteroides* and *Bifidobacterium*; dysbiosis impacts metabolism.	Early studies identified increased F/B and reduced *Bacteroides/Bifidobacterium*, supporting metabolic impact despite methodological variability.	Older, small, and heterogeneous primary studies; limited longitudinal/interventional evidence; potential publication bias.
Burananat T. et al. [97], 2025	Observational study (pediatric populations)	To evaluate microbiota, F/B ratio, and metabolic risk in obesity severity.	Variable *Actinobacteriota* and *Bacteroidota* abundance, altered F/B ratio; dysbiosis linked to metabolic risk.	Variations in *Actinobacteriota*/*Bacteroidota* balance with obesity severity suggest community composition, not single taxa, determines metabolic risk.	Observational design with heterogeneous cohorts and methods; residual confounding (diet, antibiotics, puberty) and limited external validity beyond study populations.
Liang C. et al. [98], 2020	Observational and in vitro/in vivo study (99 children, 5–15 years, Harbin, China)	To identify microbiota differences, F/B ratio, and test anti-obesity bacterial strains.	Decreased F/B ratio, reduced *Lactobacillus* and *Bifidobacterium,* increased *Akkermansia* in obesity; anti-obesity effects of strains in HFD mice.	Decreased *Lactobacillus* and *Bifidobacterium* with increased *Akkermansia* indicate microbial imbalance; validation in mice suggests potential causal and probiotic effects.	Human data are cross-sectional; causality inferred from mouse models may not translate to children; single-region cohort limits generalizability.
Araujo D.S. et al. [99], 2020	Observational study (46 children, 5–13 years, Korea)	To evaluate salivary microbiota, bacterial diversity, and gingival health in obese adolescents.	Reduced *Bacteroides* in salivary microbiota; no F/B ratio or SCFA data; linked to gingival inflammation.	Salivary dysbiosis mirrors gut microbial imbalance, indicating shared dietary and inflammatory drivers across body sites.	Small sample and cross-sectional design; salivary microbiota may not reflect gut; oral hygiene/dental disease and diet are potential confounders.
Li X.-M. et al. [100], 2024	Cohort study (children, 16S rRNA sequencing)	To associate childhood obesity with microbiota diversity, F/B ratio, and bacterial changes.	Decreased diversity, reduced *Bacteroides*, variable F/B ratio; dysbiosis correlated with obesity.	Consistently reduced diversity and *Bacteroides* confirm a core dysbiotic signature; variability in the F/B ratio reflects diet, geography, and methodology.	Observational cohort without intervention; 16S rRNA limits taxonomic/functional resolution; unmeasured diet/antibiotics may confound associations.
Jiang L.F. et al. [101], 2022	Observational study (school-aged children)	To associate microbiota diversity, F/B ratio, and obesity.	Decreased diversity, reduced *Bacteroides*, altered F/B ratio; dysbiosis linked to higher BMI.	Reduced microbial diversity and *Bacteroides* consistently associate with higher BMI, confirming diversity loss as a hallmark of pediatric obesity.	Cross-sectional design; potential reporting bias for lifestyle/diet; limited functional (metabolite) data.
Ismail H.M. et al. [102], 2025	Observational study (youth with type 1 diabetes and obesity)	To evaluate microbiota changes, F/B ratio, and SCFA levels in obese youth with type 1 diabetes.	Reduced *Bifidobacterium*, altered F/B ratio, and lower SCFA levels in obesity; dysbiosis linked to metabolic impact.	Type 1 diabetes with obesity intensifies dysbiosis and SCFA loss, showing endocrine–microbiota interdependence in metabolic outcomes.	Disease- and treatment-related confounding (autoimmunity, insulin therapy); cross-sectional design; generalizability limited beyond T1D.
Da Silva C.C. et al. [103], 2020	Observational study (obese children)	To analyze gastrointestinal microbiota, F/B ratio, and bacterial changes in childhood obesity.	Increased *Firmicutes*, reduced *Bifidobacterium,* higher F/B ratio; dysbiosis linked to fat accumulation.	Enrichment of *Firmicutes* and loss of *Bifidobacterium* support an energy-harvesting, pro-inflammatory microbial pattern associated with obesity.	Cross-sectional, likely modest sample; potential confounding by diet/antibiotics; emphasis on F/B ratio limits granularity.
Yuan X. et al. [104], 2021	Observational study (Chinese children and adolescents, obese with/without insulin resistance)	To compare gut microbiota in obese children with and without insulin resistance, focusing on bacterial diversity and the F/B ratio.	Lower microbial diversity, increased *Firmicutes*, and altered F/B ratio in obese children with insulin resistance; dysbiosis linked to metabolic disorders.	Dysbiosis with increased *Firmicutes* and reduced diversity suggests energy-harvesting capacity; insulin resistance modulates the microbial–metabolic relationship beyond the F/B ratio alone.	Cross-sectional comparisons cannot address directionality; potential confounding from diet, medications, and puberty status.
Carrizales-Sánchez A.K. et al. [105], 2021	Systematic review (children)	To analyze microbiota, F/B ratio, and SCFA roles in metabolic syndrome and obesity in children.	Increased *Firmicutes*, reduced *Bifidobacterium*, altered F/B ratio; SCFAs influence energy metabolism in metabolic syndrome.	Microbiota shifts (increased *Firmicutes*, decreased *Bifidobacterium*) and SCFA dysregulation underline a mechanistic link between gut metabolism and pediatric metabolic syndrome.	Heterogeneous case definitions and methods; few longitudinal/interventional pediatric studies; risk of publication and language bias.
Moran-Ramos S. et al. [106], 2020	Population-based study (children and early adolescents)	To evaluate environmental/intrinsic factors shaping microbiota, F/B ratio, and metabolic health.	Increased *Firmicutes*, reduced *Bifidobacterium*, altered F/B ratio; low-fiber diet linked to dysbiosis and metabolic risk.	Low-fiber diet and lifestyle factors appear central to microbiota alteration, emphasizing environmental modulation of obesity-related dysbiosis.	Predominantly cross-sectional associations; diet largely self-reported; socioeconomic and geographic factors may confound effects.
Wang L. et al. [107], 2024	Observational study (children with obesity and precocious puberty)	To analyze microbiota changes, F/B ratio, and bacterial diversity in obesity and precocious puberty.	Increased *Firmicutes*, decreased *Bacteroides*, altered F/B ratio; dysbiosis linked to metabolic risk and precocious puberty.	Altered F/B ratio and reduced *Bacteroides* in obese children with precocious puberty suggest a microbiome–endocrine axis influencing early maturation.	Cross-sectional design with small subgroup sizes; hormonal status and treatment confounders; limited mechanistic assessment.
Qian Y. et al. [108], 2024	Observational study (children with obesity and precocious puberty)	To analyze microbiota influence on obesity-associated precocious puberty and F/B ratio.	Dysbiosis with reduced *Bacteroides*, altered F/B ratio; impacts hypothalamic–gonadal axis and precocious puberty.	Altered microbiota composition in obese children with precocious puberty supports a link between gut dysbiosis and hormonal regulation.	Mechanistic claims drawn from correlative data; F/B ratio is a coarse metric; lack of longitudinal follow-up/intervention.
Gallardo-Becerra L. et al. [109], 2020	Metatranscriptomic study (Mexican children with obesity/metabolic syndrome)	To define secrebiome, microbiota profile, and SCFA levels in obesity.	Reduced abundance of 9 bacteria, lower SCFA levels, altered F/B ratio; linked to metabolic syndrome.	Reduced SCFAs and bacterial abundance reveal a functional alignment between microbial metabolism and metabolic syndrome pathology.	Likely small, cross-sectional cohort; high technical variability in RNA-based assays; diet/timing of sampling may influence expression profiles.
Wei Y. et al. [110], 2021	Observational study (children, body fat distribution)	To assess microbiota composition, F/B ratio, and SCFA levels in relation to body fat distribution.	Reduced *Bacteroides* and SCFA levels (e.g., butyrate) linked to higher body fat; altered F/B ratio; dysbiosis tied to metabolic risk.	Reduced *Bacteroides* and SCFA (butyrate) levels correlate with adiposity, suggesting a metabolic dependency between fermentation capacity and fat accumulation.	Cross-sectional design; fecal SCFAs may not capture host absorption; imaging/body-fat assessment and diet variability may confound results.
Nandy D. et al. [111], 2022	Metabolomic study (2-year-old children)	To analyze butyrate levels, microbiota, and weight outcomes.	Lower butyrate levels, altered microbiota, and F/B ratio; dysbiosis linked to early obesity.	Early-life butyrate deficiency and dysbiosis precede obesity onset, highlighting a preventive window in early childhood.	Small cohort and observational design; rapid developmental/dietary changes at age 2 confound associations; limited taxonomic resolution alongside metabolomics.
Jaimes J.D. et al. [112], 2021	Metabolomic and microbiomic study (children and adolescents)	To evaluate the stool metabolome, microbiota, and SCFA levels in obesity.	Reduced SCFA levels, lower *Bacteroides*, altered F/B ratio; dysbiosis linked to metabolic risk.	Combined metabolomic and microbiome data confirm that reduced SCFAs and *Bacteroides* underpin metabolic risk in pediatric obesity.	Cross-sectional design; 1H-NMR metabolomics has limited compound coverage; multi-site variability and diet not fully controlled.
Zhang S. & Dang Y. et al. [113], 2022	Observational study	To analyze the F/B ratio as a biological parameter of microbiome structure in obesity.	Altered F/B ratio linked to dysbiosis and obesity.	The F/B ratio remains consistently altered in obesity but is context-dependent, serving as a coarse indicator rather than a diagnostic marker.	F/B ratio is an oversimplified metric sensitive to methods, age, and diet; observational evidence lacks mechanistic/causal confirmation.

**Table 2:** Interventions related to gut microbiota modulation for obesity.

Age Group	Intervention Type	Key Clinical Applications	Supporting Authors
Infants (0–2 years)	Probiotics	May reduce obesity risk through early microbiota modulation; e.g., perinatal supplementation reduces overweight up to 10 years by promoting beneficial bacteria and SCFAs. Potential for preventing metabolic complications via gut barrier enhancement.	Borka Balas et al., 2023 [188]; Petraroli et al., 2021 [95]; Bozzi Cionci et al., 2018 [190].
Infants (0–2 years)	Synbiotics	Improves intestinal function in short bowel syndrome; supports gut barrier and reduces infections, indirectly aiding metabolic health.	Bozzi Cionci et al., 2018 [190].
Children (3–12 years)	Prebiotics	Inulin supplementation (e.g., 30 g/day) increases microbiota diversity, promotes SCFA-producing bacteria (e.g., Bifidobacterium, Agathobacter), and correlates with reduced BMI Z-score and improved fat-free mass; no direct weight loss but potential for metabolic improvements. Oligofructose-enriched inulin reduces body fat, triglycerides, and inflammation.	Visuthranukul et al., 2024 [191]; Borka Balas et al., 2023 [188]; Wang et al., 2023 [182]; Koller et al., 2025 [192].
Children (3–12 years)	Probiotics	Multi-strain probiotics (e.g., Lactobacillus, Bifidobacterium) reduce BMI, cholesterol, and inflammation and improve insulin sensitivity; e.g., B. breve strains improve glucose metabolism and weight management. Reduces NAFLD severity and liver enzymes. No consistent weight loss but aids comorbidities.	Borka Balas et al., 2023 [188]; Khongtan et al., 2023 [193]; Bozzi Cionci et al., 2018 [190]; Petraroli et al., 2021 [95]; Solito et al., 2021 [137]; Koller et al., 2025 [192]; Facchin et al., 2024 [35] (butyrate example).
Children (3–12 years)	Synbiotics	Multi-strain synbiotics (e.g., with FOS) improve anthropometric indices (e.g., waist–height ratio, BMI), body composition, and microbiota (e.g., increased Bacteroidetes); reduces inflammation and metabolic risks.	Borka Balas et al., 2023 [188]; Kilic Yildirim et al., 2023 [194]; Koller et al., 2025 [192]; Petraroli et al., 2021 [95].
Children (3–12 years)	SCFAs/Gut Modulation	SCFAs (e.g., butyrate supplementation) improve glycemic control and obesity markers when combined with therapy; modulation via diet/probiotics increases SCFA levels, reduces neuroinflammation, and enhances antioxidant capacity. Altered SCFA profiles linked to dysbiosis; potential for reducing inflammation and metabolic dysfunction.	Facchin et al., 2024 [35]; Khongtan et al., 2023 [193]; Petraroli et al., 2021 [95]; Koller et al., 2025 [192].
Adolescents (13–18 years)	Prebiotics	Limited consistent effects on weight; may reduce BMI, fat, and inflammation via microbiota shifts (e.g., increased Bifidobacterium). Potential adjunct for metabolic markers.	Wang et al., 2023 [182]; Koller et al., 2025 [192].
Adolescents (13–18 years)	Probiotics	Improves insulin sensitivity, reduces BMI and waist circumference; e.g., B. breve strains enhance metabolic homeostasis and reduce inflammation. Aids NAFLD and metabolic syndrome.	Borka Balas et al., 2023 [188]; Bozzi Cionci et al., 2018 [190]; Solito et al., 2021 [137]; Petraroli et al., 2021 [95]; Koller et al., 2025 [192].
Adolescents (13–18 years)	Synbiotics	Reduces BMI Z-score, waist circumference, and anthropometric indices; modulates microbiota (e.g., lower Firmicutes/Bacteroidetes ratio) for reduced energy harvest and inflammation.	Borka Balas et al., 2023 [188]; Kilic Yildirim et al., 2023 [194]; Koller et al., 2025 [192]; Petraroli et al., 2021 [95].
Adolescents (13–18 years)	SCFAs/Gut Modulation	SCFAs modulate appetite, insulin sensitivity, and inflammation; potential for NAFLD improvement via microbiota restoration.	Petraroli et al., 2021 [95]; Koller et al., 2025 [192].
Adults (>18 years)	Prebiotics	Increases SCFA production, improves insulin sensitivity and glucose metabolism, and reduces inflammation; e.g., inulin-type fructans for T2D and metabolic syndrome.	Facchin et al., 2024 [35]; Petraroli et al., 2021 [95].
Adults (>18 years)	Probiotics	Multi-strain formulas elevate SCFAs, improve cardiometabolic health, and reduce inflammation; mixed effects on T2D.	Facchin et al., 2024 [35]; Petraroli et al., 2021 [95].
Adults (>18 years)	Synbiotics	Reduces appetite, improves metabolic profiles; potential for obesity management.	Facchin et al., 2024 [35]; Petraroli et al., 2021 [95].
Adults (>18 years)	SCFAs/Gut Modulation	Propionate/butyrate supplementation prevents weight gain, improves insulin sensitivity, and attenuates atherosclerosis; acetate shows no benefit. Gut modulation (e.g., FMT) enhances insulin sensitivity in metabolic syndrome.	Facchin et al., 2024 [35]; Petraroli et al., 2021 [95].
General/Animal	Prebiotics	In mice, oligofructose reduces permeability, inflammation, and adiposity via GLP-2; improves gut barrier and metabolic endotoxemia. Potential for obesity prevention.	Cani et al., 2009 [195]; Facchin et al., 2024 [35].
General/Animal	Probiotics	In mice, B. breve suppresses weight gain and improves lipid metabolism.	Bozzi Cionci et al., 2018 [190].
General/Animal	Synbiotics	Enhanced effects in models for metabolic health.	Facchin et al., 2024 [35].
General/Animal	SCFAs/Gut Modulation	SCFAs regulate energy homeostasis, appetite, and inflammation via receptors; higher levels associated with obesity but protective in models.	Facchin et al., 2024 [35]; Petraroli et al., 2021 [95].

## Data Availability

No new data were created or analyzed in this study. Data sharing is not applicable to this article.
