# Correction: Pînzariu et al. Gut Microbiota and Short-Chain Fatty Acids: Key Factors in Pediatric Obesity and Therapeutic Targets. *Int. J. Mol. Sci.* 2025, *26*, 11503

**DOI:** 10.3390/ijms27125378

**Published:** 2026-06-15

**Authors:** Alin Constantin Pînzariu, Sebastian Marian Leonte, Alexandra Gabriela Trofin, Laura Mihaela Trandafir, Mihaela Moscalu, Lorena Mihaela Manole, Roxana Moscalu, Cristina Iuliana Lazăr, Luminita Georgeta Confederat, Vlad Ionuț Vlăsceanu, Radu Petru Soroceanu, Daniel Vasile Timofte, Dragomir Nicolae Șerban, Ionela Lăcrămioara Șerban

**Affiliations:** 1 Grigore T. Popa University of Medicine and Pharmacy Iasi, 700115 Iasi, Romania; alin.pinzariu@umfiasi.ro (A.C.P.); mg-rom-33565@students.umfiasi.ro (S.M.L.); mg-rom-31141@students.umfiasi.ro (A.G.T.); lorena.manole@umfiasi.ro (L.M.M.); crislazar@ymail.com (C.I.L.); confederat.luminita@email.umfiasi.ro (L.G.C.); vlad-ionut.vlasceanu@umfiasi.ro (V.I.V.); petru.soroceanu@umfiasi.ro (R.P.S.); daniel.timofte@umfiasi.ro (D.V.T.); dragomir.serban@umfiasi.ro (D.N.Ș.); ionela.serban@umfiasi.ro (I.L.Ș.); 2 Blond McIndoe Laboratories, Division of Cell Matrix Biology and Regenerative Medicine, School of Biological Sciences, Faculty of Biology, Medicine and Health, The University of Manchester, Manchester Academic Health Science Centre, Stopford Building, Manchester M13 9PL, UK; roxana.moscalu@postgrad.manchester.ac.uk

In the original publication [1], the funder Grigore T. Popa University of Medicine and Pharmacy Iasi, Contract No. 10.066/15.05.2025 to Alin Constantin Pînzariu was not included. The authors state that the scientific conclusions are unaffected. This correction was approved by the Academic Editor. The original publication has also been updated.
